# Sexual Dimorphism Impact on the Ground Reaction Force Acting on the Mediolateral Direction During Level Walking: Hip Abductor Muscle Biomechanics and Its Correlation to GRF Moment Arm

**DOI:** 10.3389/fbioe.2022.863194

**Published:** 2022-04-25

**Authors:** Amany E. Abd-Eltawab, Mariam A. Ameer, Mohamed Ahmed Eladl, Mohamed El-Sherbiny, Hasnaa Ali Ebrahim, Dalia Mahmoud Abdelmonem Elsherbini

**Affiliations:** ^1^ Physical Therapy and Health Rehabilitation Department, Faculty of Applied Medical Sciences, Jouf University, Sakaka, Saudi Arabia; ^2^ Biomechanics Department, Faculty of Physical Therapy, Cairo University, Cairo, Egypt; ^3^ Department of Basic Medical Sciences, College of Medicine, University of Sharjah, Sharjah, United Arab Emirates; ^4^ Department of Basic Medical Sciences, College of Medicine, Almaarefa University, Riyadh, Saudi Arabia; ^5^ Department of Basic Medical Sciences, College of Medicine, Princess Nourah Bint Abdulrahman University, Riyadh, Saudi Arabia; ^6^ Department of Clinical Laboratory Sciences, College of Applied Medical Sciences, Jouf University, Sakaka, Saudi Arabia; ^7^ Department of Anatomy, Faculty of Medicine, Mansoura University, Mansoura, Egypt

**Keywords:** level walking, abductor muscles, mediolateral GRF, moment arm, sexual dimorphism

## Abstract

The female pelvis morphology represents an evolved compensation between two opposing needs: a broad pelvis enough to deliver a sizeable brained offspring while remaining narrow enough to allow for effective bipedal gait. The precise expectation of hip abductor force generation is critical in anthropological studies and experimental practice of human stride mechanics. Hip implants and surgical procedures for hip anatomy reconstruction are based on the static single-leg stance paradigm. The current work investigated the impact of sexual dimorphism on the ground reaction force (GRF) acting on the mediolateral direction during level walking, emphasizing the difference in hip abductor muscle biomechanics and its correlation to ground reaction force moment arm, R. The ground reaction force in the mediolateral direction, hip abduction and adduction moments during the gait cycle and ground reaction force moment arm, R were measured. The current study concludes that the male individuals exhibit significantly higher mass-specific mediolateral ground reaction force during level walking. In contrast, hip abductor moments/kg body weight, medialization of the trochanter, R, and hip coronal were more significant in female individuals. We conclude that increased abductor moment and medialization of the greater trochanter will increase R, hip coronal and decrease abductor moment arm, r, in female individuals, affecting the effective mechanical advantage (EMA) of hip abductors in single-limb stance during level walking.

## Introduction

Muscle forces are transmitted from a specific point to the next one, triggering each foot to exert force on the ground. The ground subsequently exerts a ground reaction force (GRF) on each foot. These forces are equivalent and opposed in magnitude and direction (Rose and Gamble, 2006). This force has three components: vertical force Fz, anteroposterior force Fx, and mediolateral force Fy (Hamill and Kathleen, 2003; Mansfield and Neumann, 2019). One of the effects of the force on the hip joint during ambulation at the single-limb support phase is the reaction force’s perpendicular component, which equals five-sixths of body weight (BW) in the coronal or frontal plane direction. Thus, the gravitational force represented by body weight, which equals its reaction force, can be divided into a vertical acting component called Wy and a horizontally acting component called Wx (Nordin and Frankel, 2001).

The stance and swing phases are the two main phases of the gait cycle. The stance phase has three subphase initial contacts occurring at about 10% of the gait cycle and loading response occurring at about 20%, where the single-limb support phase includes midstance, terminal stance, and pre-swing. It extends from 30% up to 60% of the gait cycle. Meanwhile, the swing phases comprise the initial swing, mid-swing, and terminal swing. These subphases extend from 73 to 100% of the gait cycle (Oatis, 2004). Therefore, the single-limb support phase is the least stable position of the remaining subphases because only one leg is on the ground while walking, prioritizing the variation between male and female individuals in both values of Fy and Wy.

The glutei (medius and minimus) and tensor fasciae latae make up the hip abductor muscle group in humans. The anterior part of the gluteus maximus also participates in the abduction of the hip joint through the fibers inserted into the iliotibial tract. However, it is not typically regarded as a major abductor (Drake et al., 2014). The largest abductor muscle is the gluteus medius, originating from the ilium lateral surface between the anterior and posterior gluteal lines (Clark and Haynor, 1987; Drake et al., 2014). Deep in the gluteus medius, the gluteus minimus is situated, a minor muscle attached from the ilium between the inferior and anterior gluteal lines. Both muscles end on the anterosuperior part of the greater trochanter, with more lateral positioning of the gluteus medius. The tensor fasciae latae is a minor muscle originating from the anterior superior iliac spine (ASIS) and extending into the thigh’s fascia latae, which constitutes the iliotibial tract (Drake et al., 2014). The gluteus medius exerts a phasic contraction during walking and running, initially stimulating the posterior part of the muscle in an early stance to support the head of the femur in the hip socket. In contrast, the middle and anterior parts are stimulated considerably later, commencing abduction and pelvic rotation during midstance and the second half of the stance phase (Gottschalk et al., 1989; Pandy et al., 2010). At midstance, the tensor fasciae latae is most forceful. Because of its higher vertical alignment and lateral positioning on the pelvis, the tensor has a more extended moment arm than the gluteus minimus, which increases its force involvement in pelvic stability (Gottschalk et al., 1989). The gluteus minimus stabilizes the head of the femur inside the hip socket (Beck et al., 2000). Nevertheless, functional magnetic resonance imaging conducted immediately after abduction workouts revealed enhanced muscular signal intensity, suggesting action relevant to these abduction demands (Kumagai et al., 1997). So, the hip abductor muscles work energetically to support the head of the femur in the hip socket and move the thigh during a single-leg stance while walking and running (Warrener, 2011).

The body’s center of mass (COM) is moved vertically and horizontally in humans during normal bipedal locomotion when the trunk travels over the supporting leg. Several stereotyped pelvic and lower limb motions, including inclination of the pelvis in the frontal plane by roughly 5° down toward the swing side, help reduce this displacement. This motion diminishes the transverse shift needed during the step-to-step alteration by minimizing the COM elevation at midstance and adducting the stance limb. Pelvic inclination helps lower energy expenditure while walking by limiting these vertical and horizontal movements (Warrener, 2011).

Although normal stride includes some pelvic inclination during single-limb support, extreme pelvic tilt produced by weakening or hip abductor muscles paralysis induces significant gait disorders. Individuals with the Trendelenburg signs are incapable of regulating the medial descent of the pelvis in a single leg stance (Drake et al., 2005).

**FIGURE 1:**
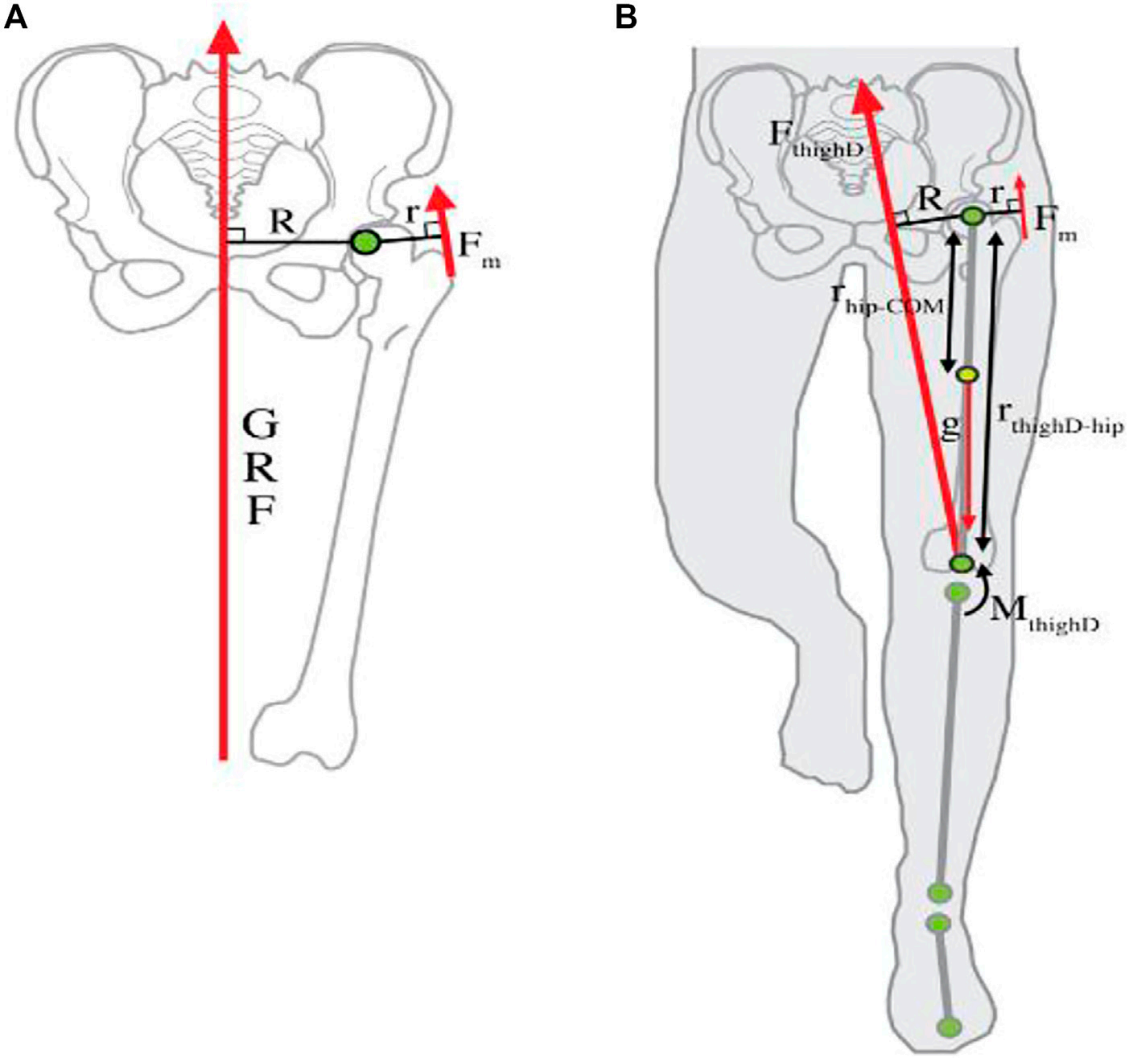
Hip abductor force production. **(A)** Typical stationary biomechanical model. **(B)** Inverse dynamics models the lower extremity (Warrener et al., 2015).

The external force trajectory crosses the medial to the center of the hip joint in the mediolateral plane, and the hip abductor muscles must create a force to keep the pelvis from sliding away from the stance leg (Arsuaga et al., 1999). Under the normal static model, abductor muscle force (F_m_) accompanies the normal value of the (GRF) external force. Effective mechanical advantage (EMA) of the hip abductor muscles is known as the ratio of the hip abductor muscle moment arm, r, to the GRF moment arm, R (Figure 1A) (Warrener et al., 2015). As this model expects that the GRF moves nearly vertically through the body’s center of gravity at the midstance phase of gait, R is expected to be nearly equal to half bi-acetabular width. EMA of the joint is presented as the ratio r/R (Warrener et al., 2015). Hip abductor forces must increase as pelvic breadth increases in order to keep the pelvis in balance and avoid extreme pelvic inclination away from the stance leg (Warrener, 2011).

Sexual variation of the human pelvis considerably results from discriminating pressures on the female pelvis, increasing the birth canal to permit the passage of a sizeable brained offspring. It compromises hip abductor EMA and leads to lower sufficient motion in female individuals compared to male individuals (Warrener et al., 2015).

While sexual dimorphism has been widely reported in numerous instances of measuring the pelvic width (LaVelle, 1995; Warrener, 2011), this study focuses on the impact of sex on the component of GRF (Fy) during the single-limb stance phase and abductor hip moment during walking. Moreover, this study is the first to determine the correlation between abductor muscles biomechanics and GRF moment Arm, R.

## Materials and Methods

### Participants and Power Analysis

Priori power analysis was implemented using statistical software (G*Power v3.1.9.4, Düsseldorf, Germany). The sample size was determined using mean and the difference between two independent means (two groups) selection in the *t*-test menu. Effect size (d) was estimated from the literature between female and male groups (Ferber et al., 2003; Rutherford and Hubley-Kozey, 2009; Warrener, 2011; Warrener et al., 2015). We assumed that the mean ± SD for the two groups (females versus males) would be (1.13 ± 0.19) and (0.82 ± 0.15) for hip abductor moment, (1.485 ± 0.20 and 1.24 ± 0.17) for hip adductor moment, and (0.0715 ± 0.01 and 0.063 ± 0.01) for ground reaction force moment arm, R. According to these assumptions, sample sizes for hip abductor moment, hip adductor moment, and ground reaction force moment arm, R, were 12, 22, and 36, respectively. Eighty percent power was attained to identify these effect sizes at an alpha level of 5%, applying the smallest effect size (d) (0.85). *t*-test design with independent two groups has sample sizes of 18 per group. The total sample of 36 achieved a power of 80% using the *t*-test with a target significance level of 0.05.

A total of 38 participants (19 males and 19 females) participated voluntarily in this study. The mean age of the participants was 19.8 ± (1.39) years, mean body mass was 72.9 ± (10.34) kg, and mean height was 174.8 ± (11.69) cm. The contributors with no musculoskeletal or mechanical problems were involved in the current research. In addition to any abnormality in the foot, posture was excluded from this study, especially history of ankle fracture that can affect foot supination or pronation. The current research work was approved by the Research Ethics Committee of Faculty of Physical Therapy of Cairo University (NO:P.T.REC/012/003543).

### Procedures

#### Qualisys Motion Capture System Preparations

**FIGURE 2:**
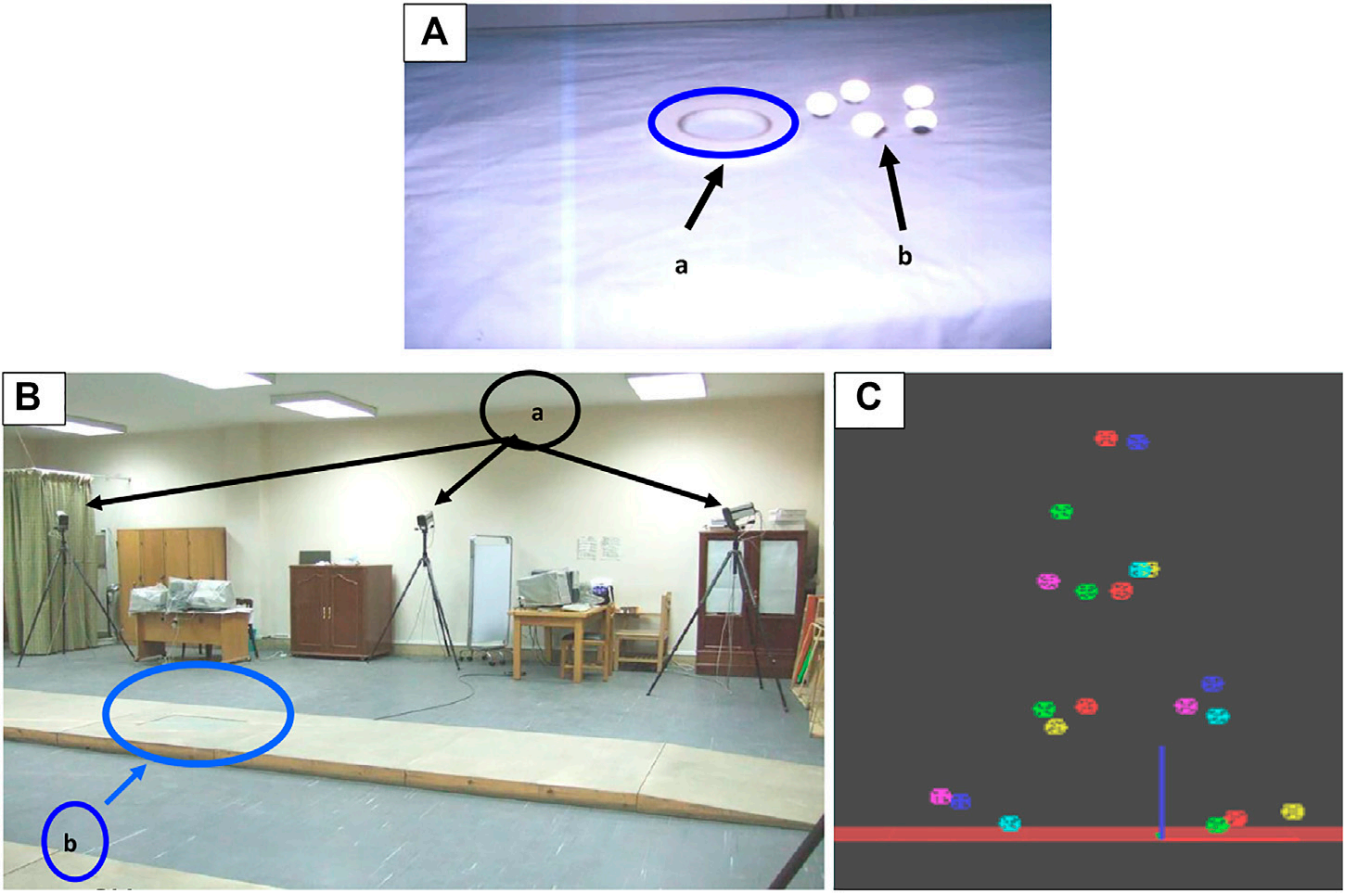
**(A)** Adhesive tapes (a) and the reflective markers (b) were used to fix the markers. **(B)** Biomechanical lab, which includes the placement of the Pro-Reflex cameras (a) in relation to the walkway and the force plate position (b) in the center of the walkway. **(C)** Exported data format, data TSV (tab-separated values).

Three-dimensional motion analysis systems (Qualisys Motion Capture System) were used in the current study in combination with a force plate component to quantify the kinetic and kinematic data. The synchronization between the force platform and motion analysis system enabled the measurement of the ground reaction forces and muscle moments in the three planes (X, Y, Z). The system structure comprised six high-velocity infrared Pro-Reflex cameras, and an AMTI (Advanced Mechanical Technology Inc., United States) force plate is impeded in the middle of a walkway. Its dimensions were 40 cm in width and 60 cm in length. The sampling rate of the plate was 120 Hz. It included four strain gauge transducers placed at the four corners of the plate. These four transducers picked up force–time data in three planes during walking. A long cable connected the force plate to a computer unit. The signals from the plate were first amplified by an internal amplifier and fed to an analog-to-digital (A/D) converter. Thus, the final output of the system was the digitization voltage values. The AMTI force plate used a coordinate oriented system in the calculation of the GRF magnitude. In such a system, the force exerted by the human body on the plate is to be measured and analyzed (Figure 2). The cameras picked up each lower limb joint position to distinguish the relative body portions during locomotion. According to the Pro-Reflex user guidebook, twenty passive reflective markers were located on specific body points (Qualisys and Gothenburg, 2011; Elhafez et al., 2019). These specific sites were 1. right and left shoulders, 2. the 12th thoracic vertebrae, 3. sacrum, 4. right and left anterior superior iliac spines, 5. right and left greater trochanters, 6. right and left suprapatellar regions, 7. right and left knee joint lines, 8. right and left tibial tuberosities, 9. right and left ankle joints, and 10. right and left heels and toes (between the 2nd and 3rd metatarsals). Double-sided adhesive tape was used to place all markers on the skin. The Qualisys system was adjusted prior to picking up the walking parameters to enable each of the six cameras to detect the locations of the reflective markers in the walkway’s path field (Senior, 2004). Each subject was asked to stand barefoot in front of the walkway and was guided to walk normally whenever possible and not target the force plate during walking. The foot placement on the force plate detected when the whole foot was located on the platform. The data were collected from three walking attempts, and the mean was calculated.

### Data Analysis

The computer software used consisted of three programs: Q trac, Q view, and Q gait. Q trac software is used to capture the three-dimensional motion of the body parts. The time of capture should be fed into the software. After capture, the software processes data and gives the three-dimensional data of each marker position. Q view software is used to view the captured data after processing. Then it is used to identify the names of the markers and to export the identified marker names as TSV (tab-separated value) format. Finally, the selected data (one complete gait cycle in addition to another 25%) of the next cycle are exported to another software (Q gait). Q gait software enables the exported data format (data. TSV) to be manipulated so that the angle can be measured and calculated in the 3 axes of motion, X, Y, and Z. Thus, the computer software provides an angle–time plot of the marker that was used to analyze the kinematics and kinetic parameters. Q Trac software recorded the right hip abduction and adduction angles. Any force exerted on the force platform was transmitted through the force transducers. The vertical GRF vector (W) can be resolute into a vertical component (Wy, which equals W. sin 90) during single limb support standing and a horizontal component (Wx, which equals W. cos 90). Thus, (Wy) equals (W) that equals 5/6 B.W (Nordin and Frankel, 2001). So, the vertical component force of the body weight equaled five-sixths of the body weight acting on the hip joint of each of male and female participants during level walking at the single-limb support. Ground reaction force data were filtered using a low-pass, fourth-order Butterworth filter, with a cutoff frequency of 10 Hz. The kinematic data were also digitally filtered using a low-pass, fourth-order Butterworth filter, with a cutoff frequency of 6 Hz (Robertson and Dowling, 2003).

The stance phase was the time from initial contact to final lift-off from the force plate by the leading limb. Data means of 5 sequential trials for each participant were detected for the analysis of the kinetic data.

The following kinetic and kinematic parameters were collected for both male and female individuals: the ankle inversion/eversion angle. The GRF was measured in the mediolateral (Fy) direction, calculated in Newton. The hip abduction and adduction moments were measured during the gait cycle. The kinematic parameters of angular displacement of hip abduction and adduction angle were also measured. The GRF moment arm, R, was calculated at the hip in the mediolateral plane. The outside force vector moves medially to the center of the hip joint, and the hip abductor muscles must yield force to avoid the pelvis from falling away from the supported limb. The needed muscular force is detected by the amount of the external force, here defined as the GRF, and the lengths of the GRF moment arm and the hip abductor muscles moment arm (Warrener, 2011).

$$\mathrm{GRF}\;\times \;\mathrm{R=}F_{m}\;\mathrm{\times r,}$$ (1)

where GRF is the vertical component force of the body weight equals five-sixths of the body weight acting on the hip joint (Wy), R is the moment arm of the GRF vector, r is the moment arm of the hip abductor muscles, and Fm is the force of the hip abductor muscles.

Abductor moment = internal torque = the product of the magnitude of the force and the vertical distance to its line of action (Chockalingam et al., 2016; Neumann, 2010):

$$\mathrm{Abductor}\;\mathrm{moment}=F_{m}\;\times \;\mathrm{r,}$$ (2)

$$R=\frac{Abductor\;moment}{GRF\;\left(Wy\right)}.$$ (3)

#### Statistical Analysis

All the previous output data were analyzed using GraphPad Prism version 9. Independent t-tests were applied to find group variances, and squared eta (*η2*) was considered as the effect size. The significance level was established at the 0.05 level of significance. Association between (R) and kinetic measures in both sexes was examined using a linear regression model with a generalized estimating equation (GEE) correction. The coefficient of determination (*r* ^2^) and analysis of variance of regression from each model was inspected, along with the exclusion of outliers. The inter-correlations among and between the independent and dependent variables of interest were evaluated. The data are normalized, and any extremes are excluded. The normality and homogeneity of variance were statistically tested before using the parametric assumption.

## Results

### Ankle Inversion–Eversion Angle

**FIGURE 3:**
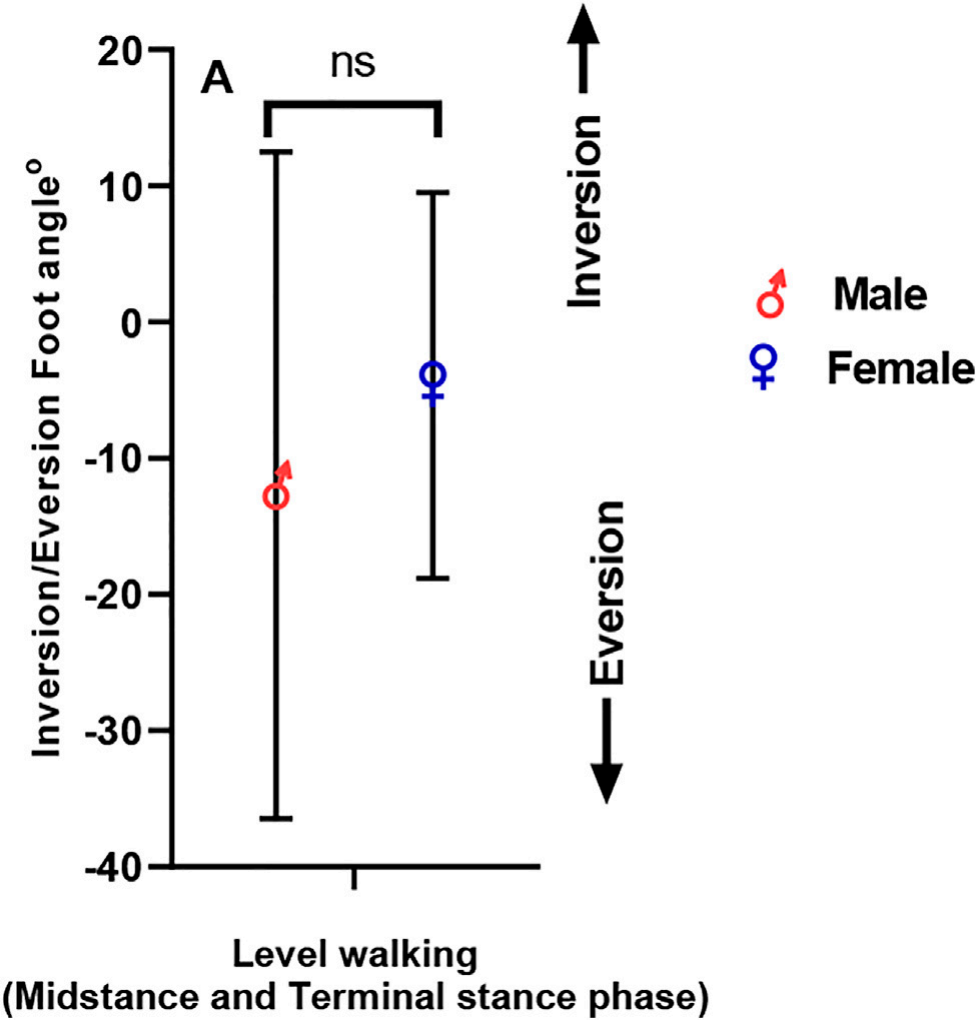
Inversion/eversion foot angle in male and female participants.

Figure 3 demonstrates the ankle inversion–eversion angle in both male and female individuals. Male individuals exhibited greater eversion than female individuals, which is insignificant (positive = inversion, negative = eversion) during level walking occurring in the frontal plane with small effect size of sex (male = −11.99 ± 24.49°, female = −4.63 ± 14.16°, *η* ^*2*^ = 0.03; *p =0.26*).

### Mediolateral (Fy) GRF

**FIGURE 4:**
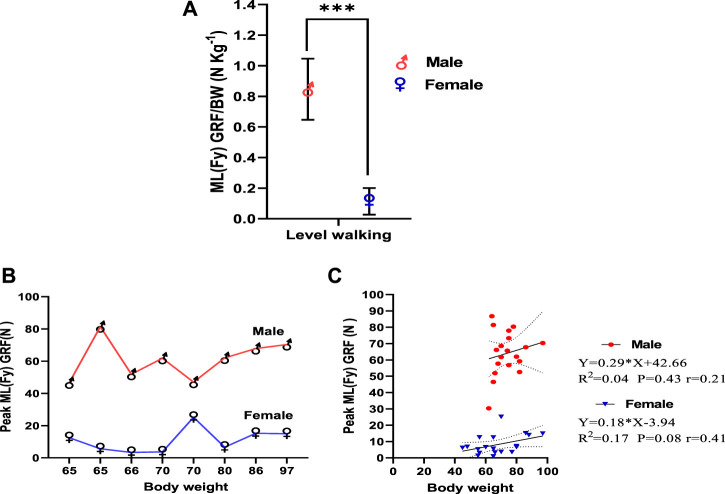
**(A)** Mediolateral force value (Fy) in male and female participants. **(B)** Inter-subject variation in mediolateral force value (Fy) in male and female participants by weight. **(C)** Linear regression of body weight versus peak ML GRF in midstance and terminal stance phases of level walking.

Figure 4 illustrates male and female mediolateral force values (Fy). Male and female individuals show significant variation in mediolateral GRF/body weight during level walking with a significant effect on size in both genders (male = 0.85 ± 0.20 N kg-1, female = 0.11 ± 0.08 N kg-1, η2 = 0.83; *p* < 0.001) (Figure 4A). Inter-subject variation was high in both male and female individuals. When comparing mediolateral GRF between male and female participants, variations in GRF by weight up to 80 Kg were observed. However, after 80 kg, the GRF increased by increasing weight in male and female individuals (Figure 4B). As indicated by linear regression in male and female individuals, this was statistically insignificant (Figure 4C). The correlation between body weight and peak mediolateral GRF was stronger in female participants than male participants. In female individuals, body weight explains 17% of the mediolateral GRF variation during level walking (*r* = 0.41; *p* = 0.08), while only 4% of the mediolateral GRF variation in male individuals (*r* = 0.21; *p* = 0.43).

### Abductor/Adductor Moment, Angle, and Greater Trochanter Displacement in the Coronal Plane

**FIGURE 5:**
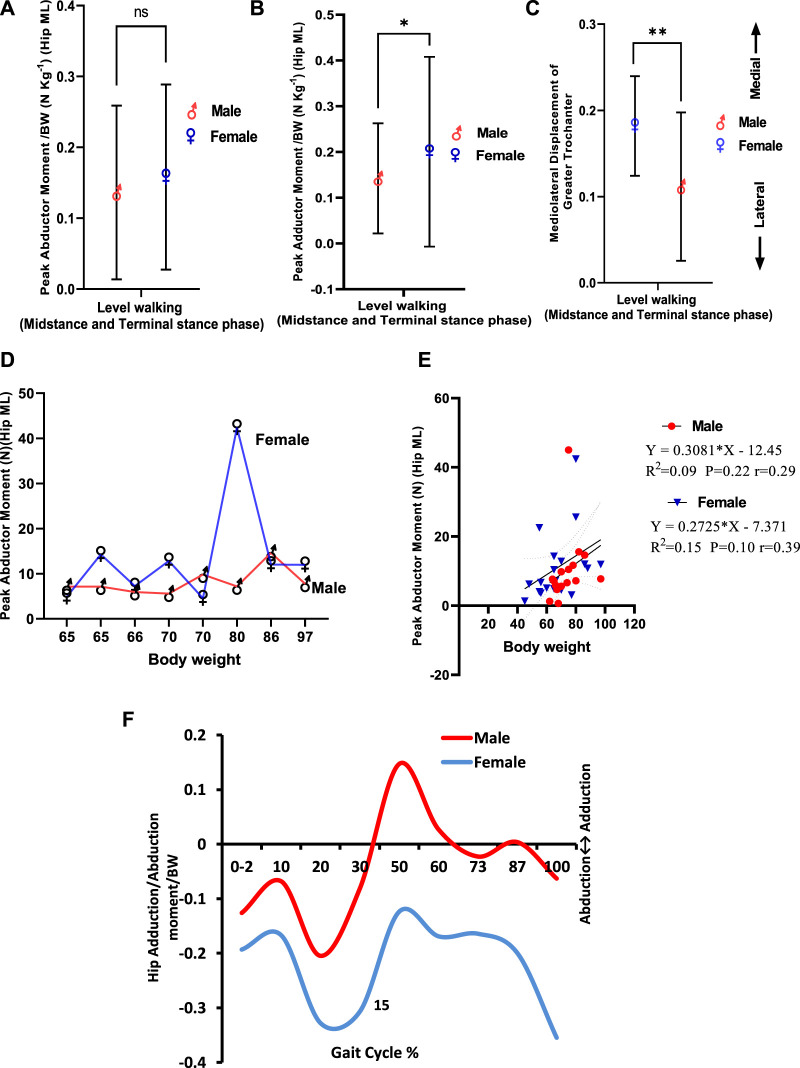
**(A)** Peak abductor moment/BW (NKg-1) in male and female participants. **(B)** Peak adductor moment/BW (NKg-1) in male and female participants. **(C)** Greater trochanter displacement in the coronal plane. **(D)** Inter-subject variation in peak abductor moment in male and female participants by weight. **(E)** Linear regression of body weight versus peak abductor moment in midstance and terminal stance phases of level walking. **(F)** Peak adductor/abductor moment/BW (NKg-1) in male and female participants during the gait cycle in level walking.

Normal walking is described by a short and adjustable adduction torque at the hip joint in the frontal plane when the body center of gravity is dragged over the stance leg. This torque quickly changed to an abduction torque, in which the lower limb exerts a lateral push on the ground’s surface, creating a medial GRF throughout the stance phase. Male and female individuals differ in mass-specific hip abductor moment during level walking (midstance and terminal stance phases) in which hip abductor moment per kilogram body weight seemed to be higher in female individuals but insignificant and showed a small size effect (male = 0.14 ± 0.12 N kg-1, female = 0.16 ± 0.13 N kg-1; η2 = 0.01; *p* = 0.80) (Figure 5A). Hip adductor moment/Kg is significantly higher in female individuals than in male individuals with moderate effect size (male = 0.14 ± 0.12 N kg-1, female = 0.20 ± 0.18 N kg-1, *η* 2 = 0.06; *p* < 0.05) (Figure 5B). Figure 5C demonstrates that the greater trochanter is more displaced medially in female individuals (0.11 ± 0.08°) than in male individuals (0.18 ± 0.06°), which is significant (*p* < 0.01), and η2 = 0.19 reflects a large effect size on gender. When comparing abductor moments between male and female individuals, interpersonal variations were observed by weight, but generally, the moment was greater in female individuals (Figure 5D). This was statistically insignificant as indicated by linear regression in male and female individuals (Figure 5E). The correlation between body weight and hip abductor moments was stronger in female individuals than in male individuals. In female individuals, body weight explains 15% of the variation in abductor moments during level walking (*r = 0.39*; *p* = 0.10), while in male participants, that explains only 9% of the variation in abductor moments (*r* = 0.29; *p* = 0.22). At the hip joint, abduction moments in the midstance phase of the gait cycle (10–30%) in female individuals are greater than that in male participants. It was also observed that abductor moment in female participants prevails during terminal stance (30–50%) and pre-swing (50–60%) phases of the gait cycle. In contrast, adductor moments in male participants prevail during these phases (Figure 5F).

**FIGURE 6:**
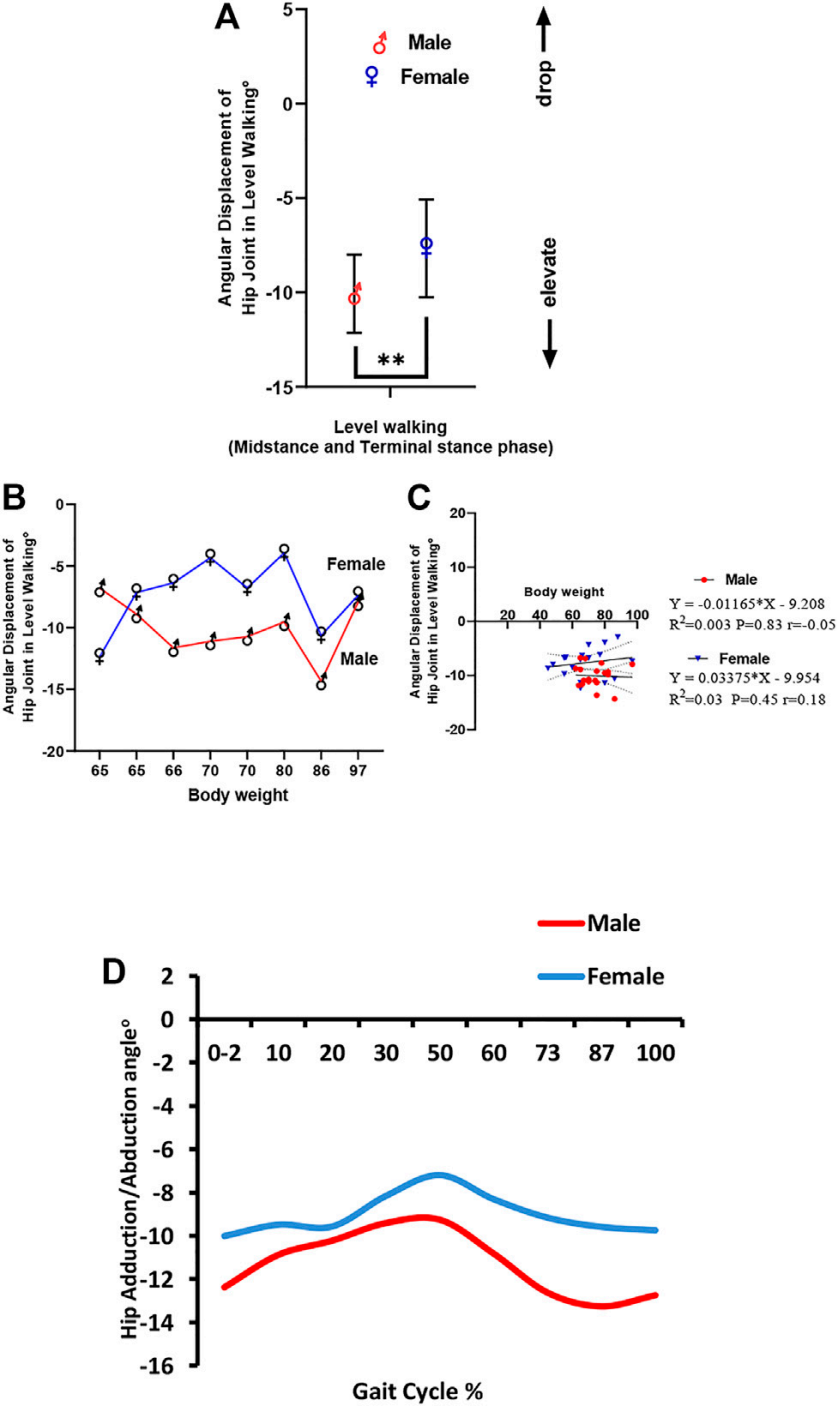
**(A)** Angular translation of the hip joint in the coronal plane in male and female participants. **(B)** Inter-subject variation in angular displacement of the hip joint in the coronal plane in male and female participants by weight. **(C)** Linear regression of body weight versus angular displacement of the hip joint during level walking. **(D)** Hip adductor/abductor angle in male and female participants during the gait cycle in level walking.

One of the key functions of the hip abductors is to control the pelvic tilt during walking. Positive magnitudes indicate inclined pelvis away from the stance limb, and negative values specify the raising of the pelvis to the stance leg. Male and female participants vary in pelvic tilting during level walking (midstance and terminal stance phases) in which pelvic inclination tends to be greater in male individuals toward abduction than in female individuals toward adduction, which is significant and shows a large effect size for sex (male = −10.07 ± 2.08°, female = −7.67 ± 2.59°, *η* ^*2*^ = 0.22; *p < 0.05*) (Figure 6A). When comparing pelvic inclination between male and female individuals, variation by weight as the pelvic angle in a female individual tends to decrease up to 80 Kg body weight, while in male individuals, it tends to increase up to 80 Kg. However, after 80 Kg, it showed a sharp increase in both male and female individuals (Figure 6B). This was statistically insignificant, as indicated by linear regression in male and female individuals (Figure 6C). The correlation between body weight and pelvic angle was more significant in female individuals than in male individuals. In female participants, body weight explained 3% of the variation in pelvic angle during level walking (*r* = 0.18; *p* = 0.45), while in male participants, only 0.3% of the variation in the pelvic angle (*r* = 0.5; *p* = 0.83) was explained. At the hip joint, pelvic inclination in all phases of the gait cycle in male individuals was greater than that in female individuals. It was also observed that pelvic inclination decreased in both male and female participants during terminal stance (30–50%) and pre-swing (50–60%) phases of the gait cycle and started to increase again during the swing phase (Figure 6D).

### Correlation Between Mediolateral GRF and Ground Reaction Force Moment Arm, R

**FIGURE 7:**
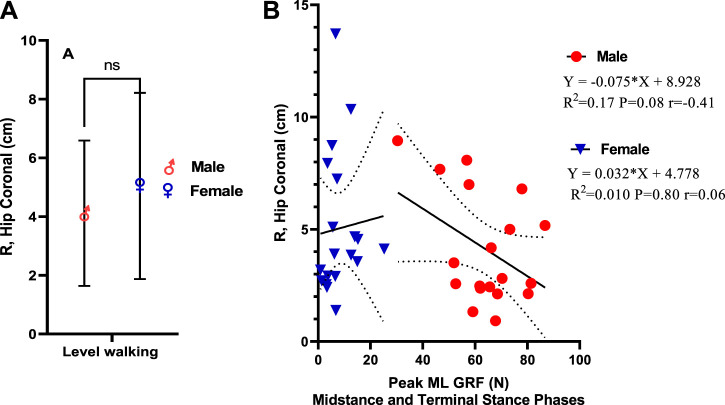
**(A)** Ground reaction force moment arm, R. **(B)** Linear regression of mediolateral GRF versus ground reaction force moment arm, R.

The mediolateral component of GRF leads to the resolute force vector to move away medially from the stance side during single-leg support. Figure 7A demonstrates that female individuals have a greater R, hip coronal vector (5.04 cm ± 3.170) than male individuals (4.11 cm ± 2.48), which was insignificant with a small effect size for gender (*η* ^*2*^ = 0.03; *p* = 0.30). Figure 7B demonstrates a weak positive correlation between ML GRF and R, hip coronal (*r* = 0.06) compared to male individuals, which shows an intermediate negative correlation (*r* = −0.41). This was statistically insignificant, as indicated by linear regression in male and female individuals. In female individuals, ML GRF explains 1% of the variation in R, hip coronal during level walking (*p* = 0.80), while in male individuals, it explains 17% of the variation (*p* = 0.08).

### Correlation Between Abductor Moment, Greater Trochanter Displacement, and Ground Reaction Force Moment Arm, R

**FIGURE 8:**
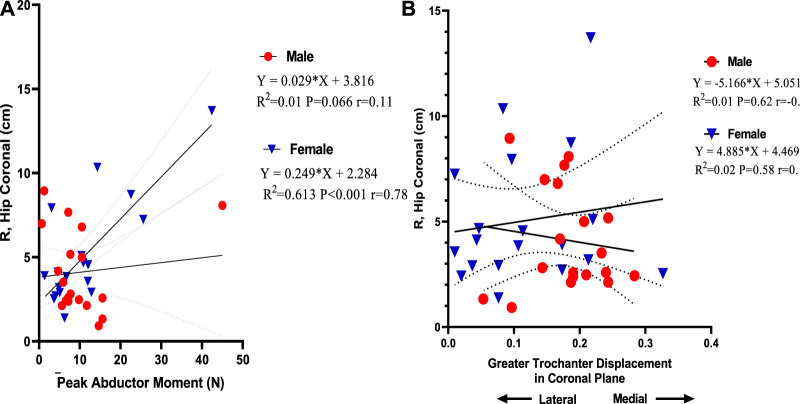
**(A)** Linear regression of peak abductor moment versus ground reaction force moment arm, R. **(B)** Linear regression of greater trochanter displacement versus ground reaction force moment arm, R.

Figure 8A demonstrates a strong positive correlation between R, hip coronal vector, and peak abductor moment in female participants (*r* = 0.78) compared to male participants. This was statistically significant, as indicated by linear regression in female individuals. In female participants, peak abductor moment explains 61% of the variation in R, hip coronal in level walking (*p* < 0.001), while in male participants, it explains 1% of the variation (*p* = 0.07). Figure 8B demonstrates a direct correlation between R, hip coronal vector, and medial greater trochanter displacement in the coronal plane in female individuals (*r* = 0.14), which is positively correlated when compared with male individuals, which showed a negative correlation (*r* = −0.12). This means a medial displacement of the greater trochanter increases R, hip coronal vector. This was statistically insignificant, as indicated by linear regression in male and female individuals. In female participants, hip medial displacement explains 2% of the variation in R, hip coronal during level walking (*p* = 0.62), while in male individuals, it explains 1%.

## Discussion

Sexual dimorphism of the human pelvis is commonly defined as the consequence of opposing selection forces on the female pelvis functioning to widen the female birth canal to facilitate passage of sizeable-brained newborn while minimizing the width of the pelvis for adequate mobility (Rosenberg and Trevathan, 2002). A previous study has revealed significant kinematic variances among men and women during walking, widely thought to be caused by dimorphic elements of skeletal structure (Cho et al., 2004).

Normal male and female individuals achieve a variety of single-leg procedures differently, including single-leg landing (Jacobs et al., 2007; Schmitz et al., 2007) and single-leg step down (Earl et al., 2007). While these research studies have established kinematic differences between male and female individuals in running and squatting, it was indistinct how to force creation, and how joint mechanics had been affected by these alterations during walking. Moreover, few research studies have tested gender-related variances in lower extremity mechanics during walking.

The key movement of the ankle joint complex is plantar–dorsiflexion (in the sagittal plane), ab-/adduction (in the transverse plane), and inversion–eversion (in the frontal) (Zwipp and Randt, 1994). Combinations of these movements across both the subtalar and tibiotalar joints generate three-dimensional motions called supination and pronation. Both terms describe the position of the plantar surface of the foot (Nordin and Frankel, 2001). The sole of the foot faces medially during supination as a result of a combination of plantar flexion, inversion, and adduction. In pronation, dorsiflexion, eversion, and abduction act to turn the sole laterally (Brockett and Chapman, 2016). Foot pronation, on the other hand, is difficult to quantify due to its three-dimensional nature, and there is significant variation in the orientation of the subtalar axis across subjects and joint positions (Kirby, 2001). Foot eversion–inversion is frequently used to estimate foot pronation–supination because it is largely independent of motions in other joints, making it less prone to errors (Perry and Lafortune, 1995), and we followed this in our study, which showed insignificant greater eversion in male individuals than in female individuals with small effect size during level walking. This result was consistent with a previous study that postulated the insignificant sex variation of supination and pronation in range of motion relating this to nearly small effect sizes, which led to greater differences mainly in running than in walking (Bruening et al., 2020).

In our study, male participants exhibited significantly higher mass-specific mediolateral GRF during level walking than female participants. There was also higher inter-subject variation in both male and female participants. The correlation between body weight and peak mediolateral GRF was stronger in female than in male participants.

Warrener (2011) reported that male individuals have higher mass-specific mediolateral GRF during slow walking than female individuals. However, women were considered to have higher mediolateral GRF/Kg body weight at the highest walking velocity and during running with a high inter-subject variation. This increase in male participants may be attributed to the difference in gender features and habits (Cho et al., 2004). The morphology and metabolites necessary for muscular contraction affect both genders (Aniansson et al., 1986), revealing that the compressive force generated by the male muscle while walking was dependent on fiber distribution and body cell mass for a greater reaction force of the lower limbs joint of male individuals than female individuals. Another factor can be added—foot eversion (pronation), which is common in male individuals, but this was insignificant. This could be attributed to that our study focuses on variation during walking not running. Morley et al. (2010) reported that upon the relative times of occurrence, the peak medial GRF occurred closer to the peak eversion than the peak lateral GRF. Matsusaka (1986) previously explained that when the lateral component force was larger, the pronation of the foot was smaller. During the midstance phase, the peroneus longus was active, and the tibialis posterior activity subsided earlier than in the smaller component. It is proposed that the peroneus longus must be active during the midstance phase to prevent the lower leg from inclining medially over the stationary foot. Furthermore, it is considered that the peroneus longus works to stabilize the head of the first metatarsal. Reinschmidt and Nigg (2000) previously demonstrated that mediolateral GRF are not only determined by rear foot motion, which is just one component of the movements that occur in the frontal plane but also by other intrinsic (e.g., height and stiffness of the medial longitudinal arch of the runner’s foot) and functional (e.g., subject specific movement coupling between foot and leg) factors during running.

Regarding hip abductor moments/Kg BW, female individuals exhibited greater abductor moments/Kg BW. By comparing abductor moments between male and female individuals, interpersonal variations were observed by weight, but generally, the moment was greater in female individuals but statistically insignificant. The correlation between body weight and hip abductor moments was stronger in female individuals than in male individuals. At the hip joint, abductor moments/Kg BW in the midstance phase, terminal stance, and pre-swing phases of the gait cycle are greater in female individuals than in male individuals. On the contrary, adductor moments in the male individual prevail during these phases.

Warrener et al. (2015) stated that hip abductor moments/kg body weight is higher in female individuals at all speeds. Warrener (2011) explained that according to the hypothesis, as pelvic breadth increases, forces exerted by the hip abductor must also be enhanced to keep the pelvis in balance to avoid extreme pelvic tilting away from the stance limb. In the current study, increasing hip abductor moment should not be considered an index of increased abductor muscle force. Rutherford and Hubley-Kozey (2009) stated that the strength characteristics of the hip abductor muscles have no direct effect on the coronal plane hip moments of force during walking. Although the strength of the hip abductor muscles is one of the several factors that could affect the moments of the muscle forces about the hip in the coronal plane, gait speed and subject body weight were found to be the main effectors in their study. This was similarly observed in the current study by the correlation between body weight and hip abductor moments in female individuals compared to male individuals. They also indicated that the tension of the hip abductor muscles is not associated with the peak value of this moment in normal individuals. They proposed that other muscles (gluteus maximus, tensor fascia lata) or passive support structures (iliotibial tract) may have exerted additional torque impacts that were not assessed in the present work.

Another interesting finding in our study is that female individuals have a greater R, hip coronal vector, and a strong positive correlation between R, hip coronal vector, and peak abductor moment than male individuals. Warrener (2011) reported that R in the frontal plane is greater in female individuals than in male individuals at each walking and running velocity. This was consistent with our results. There is a weak positive correlation between mediolateral GRF and R, hip coronal in female individuals compared to male individuals, in which increased ML GRF leads to an increase in R, hip coronal, and vice versa. In our research, male participants showed higher mass-specific ML GRF during slow walking than female participants. They were inversely correlated with R, hip coronal, but previous research conducted by Warrener (2011) reported that male individuals have higher mass-specific mediolateral GRF during slow walking than female individuals. However, female individuals seem to have considerably greater mass-specific mediolateral GRF at the maximum walking velocity and during running. So, we suggest that a weak positive correlation with R, hip coronal on the reverse of inverse correlation in male individuals might be a contributing factor in increased R, hip coronal in female individuals. Also, a strong correlation between hip abductor moments and R in the frontal plane is an interesting finding as our research finds that increasing abductor moment increases R-value in female individuals. Each muscle group’s effective mechanical advantage (EMA) is calculated as (r/R). So in the current research, we proposed that increased R will decrease EMA in women. Warrener et al. (2015) stated that men and women are sexually dimorphic on the other side of the pelvis, mainly those primarily associated with obstetrics. Female individuals have larger bispinous, mediolateral pelvic outlet dimensions and greater actual biacetabular width (quantified as the distance from the innermost aspect of the right and left acetabula). Rosenberg and Trevathan (2002) demonstrated that the obstetrical dilemma expects that greater pelvic breadth in female bispinous s accompanying the burdens of birthing sizeable-brained infants negatively affects hip abductor EMA and leads to less effective walking in female individuals than male individuals. This is consistent with our hypothesis as increased ML GRF in male individuals leads to a decrease in R, hip coronal, which leads to increased EMA in male individuals compared to female individuals. We suggest that the increasing effect of medial displacement of the greater trochanter on R, the hip coronal vector is another contributing factor in decreased abductor EMA. Iglič et al. (1995) concluded that lateral movement of the greater trochanter is helpful because it reduces the level of the hip joint contact force and the needed resultant muscle hip force. Medialization of the greater trochanter is biomechanically unfavorable because it concurrently increases needed force and R, which is the most disparaging mixture. Therefore, hypothetically the medialization of the greater trochanter should always be avoided. Henderson et al. (2011) concluded that the hip abductor moment arm (r) is influenced by frontal plane motion changes and the insertion location of the abductor muscles. As a muscle’s moment arm is the length of a line drawn vertical to the line of action and intersecting the joint center of rotation, superior and lateral movement of the greater trochanter will change the abductor muscle insertion in a direction that tends to increase moment arm. On the contrary, inferior or medial movement will move the insertion in a direction that decreases moment, suggesting that greater trochanter medialization will decrease the abductor moment arm (r) in female individuals and subsequently decrease EMA as we mentioned before that EMA = *r*/R.

Regarding the hip abductor/adductor angle, it was observed that it decreased in both male and female individuals during terminal stance and pre-swing phases of the gait cycle and started to increase again during the swing phase. However, the values in female participants were toward the positive value (adductors) as compared with male participants, which was more negative (abductors). A significant increase in hip adductor moment compared with hip abductor moment was consistently seen in our study. Female participants have been mainly observed to walk and run with higher hip adduction (Graci et al., 2012) in addition to internal rotation of the femur and increasing the angle of the knee valgus (Ferber et al., 2003; Cho et al., 2004). These alterations are supposed to lead to the greater occurrence of patellofemoral pain syndrome and iliotibial band injuries in female individuals compared to male individuals (Taunton et al., 2002; Prins and Van der Wurff, 2009).

## Conclusion and Recommendations

Adduction/abduction angle analysis revealed the predominance of adduction in female individuals, which was supported by a significant increase in hip adductor moment resembling hip abductor moment. This increase in abductor moment and medialization of the greater trochanter will increase moment arm, R, hip coronal in female individuals that will affect effective mechanical advantage of hip abductors in single-limb stance during level walking. Thus, training exercises aimed to increase the strength of abductor muscles and decrease adduction in women during a walk and run should be recommended to decrease the occurrence of patellofemoral pain syndrome and iliotibial band injuries.

## Data Availability

The original contributions presented in the study are included in the article/Supplementary Material, further inquiries can be directed to the corresponding authors.

## References

[B1] Aniansson A. Hedberg M. Henning G.-B. Grimby G. (1986). Muscle Morphology, Enzymatic Activity, and Muscle Strength in Elderly Men: A Follow-Up Study. Muscle Nerve 9, 585–591. 10.1002/mus.880090702 3762579

[B2] Arsuaga J.-L. Lorenzo C. Carretero J.-M. Gracia A. Martínez I. García N. (1999). A Complete Human Pelvis from the Middle Pleistocene of Spain. Nature 399, 255–258. 10.1038/20430 10353247

[B3] Beck M. Sledge J. B. Gautier E. Dora C. F. Ganz R. (2000). The Anatomy and Function of the Gluteus Minimus Muscle. The J. Bone Jt. Surg. Br. volumeBritish 82-B, 358–363. 10.1302/0301-620x.82b3.0820358 10813169

[B4] Brockett C. L. Chapman G. J. (2016). Biomechanics of the Ankle. Orthopaedics and trauma 30, 232–238. 10.1016/j.mporth.2016.04.015 27594929 PMC4994968

[B5] Bruening D. A. Baird A. R. Weaver K. J. Rasmussen A. T. (2020). Whole Body Kinematic Sex Differences Persist across Non-dimensional Gait Speeds. Plos one 15, e0237449. 10.1371/journal.pone.0237449 32817696 PMC7440644

[B6] Cho S. H. Park J. M. Kwon O. Y. (2004). Gender Differences in Three Dimensional Gait Analysis Data from 98 Healthy Korean Adults. Clin. Biomech. 19, 145–152. 10.1016/j.clinbiomech.2003.10.003 14967577

[B7] Clark J. M. Haynor D. R. (1987). Anatomy of the Abductor Muscles of the Hip as Studied by Computed Tomography. J. Bone Jt. Surg. 69, 1021–1031. 10.2106/00004623-198769070-00010 3654693

[B8] Drake R. Drake R. L. Gray H. Vogl W. Mitchell A. W. (2005). Gray's Anatomy for Students. Philadelphia: Elsevier/Churchill Livingstone.

[B31] Drake R. L. Vogl W. Mitchell A. W. M. Gray H. Gray H. (2014). Gray’s Anatomy for Students. Philadelphia, Pennsylvania: Churchill Livingstone/Elsevier.

[B9] Earl J. E. Monteiro S. K. Snyder K. R. (2007). Differences in Lower Extremity Kinematics between a Bilateral Drop-Vertical Jump and a Single-Leg Step-Down. J. Orthop. Sports Phys. Ther. 37, 245–252. 10.2519/jospt.2007.2202 17549953

[B10] Elhafez S. M. Ashour A. A. Elhafez N. M. Elhafez G. M. Abdelmohsen A. M. (2019). Percentage Contribution of Lower Limb Moments to Vertical Ground Reaction Force in normal Gait. J. chiropractic Med. 18, 90–96. 10.1016/j.jcm.2018.11.003 PMC6656910 31367195

[B11] Ferber R. McClay Davis I. Williams III D. S. Iii (2003). Gender Differences in Lower Extremity Mechanics during Running. Clin. Biomech. 18, 350–357. 10.1016/s0268-0033(03)00025-1 12689785

[B12] Gottschalk F. Kourosh S. Leveau B. (1989). The Functional Anatomy of Tensor Fasciae Latae and Gluteus Medius and Minimus. J. Anat. 166, 179–189. 2621137 PMC1256751

[B13] Graci V. Van Dillen L. R. Salsich G. B. (2012). Gender Differences in Trunk, Pelvis and Lower Limb Kinematics during a Single Leg Squat. Gait & Posture 36, 461–466. 10.1016/j.gaitpost.2012.04.006 22591790 PMC3407338

[B14] Hamill J. Kathleen M. (2003). Knutzen K. Biomechanical Basis of Human Movement. Philadelphia: Williams & Wilkins.

[B15] Henderson E. R. Marulanda G. A. Cheong D. Temple H. T. Letson G. D. (2011). Hip Abductor Moment Arm-Aa Mathematical Analysis for Proximal Femoral Replacement. J. Orthop. Surg. Res. 6, 6–10. 10.1186/1749-799X-6-6 21266066 PMC3247065

[B16] Iglič A. Antolič V. Srakar F. Kralj-Iglič V. Maček-Lebar A. Brajnik D. (1995). Biomechanical Study of Various Greater Trochanter Positions. Arch. orthopaedic Trauma Surg. 114, 76–78. 10.1007/BF00422829 7734237

[B17] Jacobs C. A. Uhl T. L. Mattacola C. G. Shapiro R. Rayens W. S. (2007). Hip Abductor Function and Lower Extremity landing Kinematics: Sex Differences. J. Athl Train. 42, 76–83. 17597947 PMC1896084

[B18] Kirby K. A. (2001). Subtalar Joint axis Location and Rotational Equilibrium Theory of Foot Function. J. Am. Podiatric Med. Assoc. 91, 465–487. 10.7547/87507315-91-9-465 11679628

[B19] Kumagai M. Shiba N. Higuchi F. Nishimura H. Inoue A. (1997). Functional Evaluation of Hip Abductor Muscles with Use of Magnetic Resonance Imaging. J. Orthop. Res. 15, 888–893. 10.1002/jor.1100150615 9497815

[B20] LaVelle M. (1995). Natural Selection and Developmental Sexual Variation in the Human Pelvis. Am. J. Phys. Anthropol. 98, 59–72. 10.1002/ajpa.1330980106 8579191

[B21] Mansfield P. J. Neumann D. A. (2019). Essentials of Kinesiology for the Physical Therapist Assistant E-Book. St. Louis, Missouri: Elsevier/Mosby.

[B22] Matsusaka N. (1986). Control of the Medial-Lateral Balance in Walking. Acta Orthopaedica Scand. 57, 555–559. 10.3109/17453678609014793 3577730

[B23] Morley J. B. Decker L. M. Dierks T. Blanke D. French J. Stergiou N. (2010). Effects of Varying Amounts of Pronation on the Mediolateral Ground Reaction Forces during Barefoot versus Shod Running. J. Appl. Biomech. 26, 205–214. 10.1123/jab.26.2.205 20498492

[B24] Nordin M. Frankel V. H. (2001). Basic Biomechanics of the Musculoskeletal System. Lippincott Williams & Wilkins.

[B25] Oatis C. (2004). Characteristics of normal Gait and Factors Influencing itKinesiology: The Mechanics and Pathomechanics of Human Movement. Oatis, CAPhiladelphia: PA: Lippincott Williams & Wilkins, 867–877.

[B26] Pandy M. G. Lin Y.-C. Kim H. J. (2010). Muscle Coordination of Mediolateral Balance in normal Walking. J. Biomech. 43, 2055–2064. 10.1016/j.jbiomech.2010.04.010 20451911

[B27] Perry S. Lafortune M. (1995). Influences of Inversion/eversion of the Foot upon Impact Loading during Locomotion. Clin. Biomech. 10, 253–257. 10.1016/0268-0033(95)00006-7 11415562

[B28] Prins M. R. Van der Wurff P. (2009). Females with Patellofemoral Pain Syndrome Have Weak Hip Muscles: a Systematic Review. Aust. J. Physiother. 55, 9–15. 10.1016/s0004-9514(09)70055-8 19226237

[B29] Qualisys A. Gothenburg S. (2011). Qualisys Track Manager. New York: User Manual.

[B30] Reinschmidt C. Nigg B. M. (2000). Current Issues in the Design of Running and Court Shoes. Sportverletz Sportschaden 14, 72–81. 10.1055/s-2000-7866 11081243

[B32] Robertson D. G. E. Dowling J. J. (2003). Design and Responses of Butterworth and Critically Damped Digital Filters. J. Electromyogr. Kinesiol. 13, 569–573. 10.1016/s1050-6411(03)00080-4 14573371

[B33] Rose J. Gamble G. (2006). Kinetics of normal Walking; Energetics of Walking. Human Walking. Philadelphia: Lippincott: Williams and Wilkins.

[B34] Rosenberg K. Trevathan W. (2002). Birth, Obstetrics and Human Evolution. BJOG: Intern. J. Obs Gyn 109, 1199–1206. 10.1046/j.1471-0528.2002.00010.x 12452455

[B35] Rutherford D. J. Hubley-Kozey C. (2009). Explaining the Hip Adduction Moment Variability during Gait: Implications for Hip Abductor Strengthening. Clin. Biomech. 24, 267–273. 10.1016/j.clinbiomech.2008.12.006 19136181

[B36] Schmitz R. J. Kulas A. S. Perrin D. H. Riemann B. L. Shultz S. J. (2007). Sex Differences in Lower Extremity Biomechanics during Single Leg Landings. Clin. Biomech. 22, 681–688. 10.1016/j.clinbiomech.2007.03.001 17499896

[B37] Senior D. (2004). Qualisys Track Manager: User Manual:ProReflex User Guide. Gothenburg,Sweden: Qualysis Medical AB.

[B38] Taunton J. E. Ryan M. B. Clement D. McKenzie D. C. Lloyd-Smith D. Zumbo B. (2002). A Retrospective Case-Control Analysis of 2002 Running Injuries. Br. J. Sports Med. 36, 95–101. 10.1136/bjsm.36.2.95 11916889 PMC1724490

[B39] Warrener A. G. (2011). Pelvic Shape, Hip Abductor Mechanics and Locomotor Energetics in Extinct Hominins and Modern Humans. St. Louis: Washington University.

[B40] Warrener A. G. Lewton K. L. Pontzer H. Lieberman D. E. (2015). A Wider Pelvis Does Not Increase Locomotor Cost in Humans, with Implications for the Evolution of Childbirth. PloS one 10, e0118903. 10.1371/journal.pone.0118903 25760381 PMC4356512

[B42] Zwipp H. Randt T. (1994). Ankle Joint Biomechanics. Foot Ankle Surg. 1, 21–27. 10.1016/s1268-7731(05)80052-9

